# Ruminal fermentation pattern of acidosis-induced cows fed either monensin or polyclonal antibodies preparation against several ruminal bacteria

**DOI:** 10.3389/fvets.2023.1090107

**Published:** 2023-03-20

**Authors:** Rodrigo D. L. Pacheco, Johnny M. Souza, Carolina T. Marino, João Paulo S. T. Bastos, Cyntia L. Martins, Paulo H. M. Rodrigues, Mario D. B. Arrigoni, Danilo D. Millen

**Affiliations:** ^1^Department of Breeding and Animal Nutrition, School of Veterinary Medicine and Animal Science, São Paulo State University (UNESP), Botucatu, Brazil; ^2^Department of Animal Production, College of Agricultural and Technological Sciences, São Paulo State University (UNESP), Dracena, Brazil; ^3^Embrapa Beef Cattle, Campo Grande, Brazil; ^4^School of Veterinary Medicine and Animal Science, University of São Paulo, São Paulo, Brazil

**Keywords:** additives, rumen, *Streptococcus bovis*, *Fusobacterium necrophorum*, *Lactobacillus ssp*

## Abstract

This study was designed to evaluate a spray-dried multivalent polyclonal antibody preparation (PAP) against lactate-producing bacteria as an alternative to monensin (MON) to control ruminal acidification. Holstein cows (677 ± 98 kg) fitted with ruminal cannulas were allocated in an incomplete Latin square design with two 20 days period. Cows were randomly assigned to control (CTL), PAP, or MON treatments. For each period, cows were fed a forage diet in the first 5 days (d−5 to d−1), composed of sugarcane, urea and a mineral supplement, followed by a 74% concentrate diet for 15 days (d 0 to d 14). There were no treatment main effects (*P* > 0.05) on dry matter intake (DMI) and microbial protein synthesis. However, there was a large peak (*P* < 0.01) of intake on d 0 (18.29 kg), followed by a large decline on d 1 (3.67 kg). From d2, DMI showed an increasing pattern (8.34 kg) and stabilized around d 8 (12.96 kg). Higher mean pH was measured (*P* < 0.01) in cattle-fed MON (6.06 vs. PA*P* = 5.89 and CTL = 5.91). The ruminal NH_3_-N concentration of CTL-fed cows was lower (*P* < 0.01) compared to those fed MON or PAP. The molar concentration of acetate and lactate was not affected (*P* > 0.23) by treatments, but feeding MON increased (*P* = 0.01) propionate during the first 4 days after the challenge. Feeding MON and PAP reduced (*P* = 0.01) the molar proportion of butyrate. MON was effective in controlling pH and improved ruminal fermentation of acidosis-induced cows. However, PAP was not effective in controlling acidosis. The acidosis induced by the challenge was caused by the accumulation of SCFAs. Therefore, the real conditions for evaluation of this feed additive were not reached in this experiment, since this PAP was proposed to work against lactate-producing bacteria.

## 1. Introduction

Intensive cattle management systems often include increased amounts of cereal grains in the diet to increase energy input and improve performance (1). Thus, energy-dense diets typically fed to highly productive ruminants can lead to digestive disorders, such as ruminal acidosis, in response to the rapid ruminal fermentation of starch and sugars (2). Ruminal acidosis is the most economically important metabolic disorder in intensive cattle production systems, and that exists in both acute and subacute forms (3). Ruminal acidosis has been reported as the second most common health problem affecting feedlot cattle in Brazil (1).

Sub-acute ruminal acidosis (SARA) is an ongoing problem in the dairy and feedlot sector, responsible for the onset of different pathologies, such as rumenitis, parakeratosis, laminitis, and metabolic acidosis. *Streptococcus bovis* was the major initiators of ruminal acidosis by producing lactate as the major fermentation product under low ruminal pH (4). This results in consistent economic losses in both beef and dairy industries primarily due to decreased efficiency of milk production, premature culling, and reduction in milk yield and milk fat (5, 6) as well as a reduction on efficiency and performance of beef cattle (6, 7).

In this context, ionophore antibiotics, such as monensin (MON), are widely used in feedlot diets for acidosis control by modulating rate the of ruminal fermentation reducing dry matter intake (8, 9). Moreover, this ionophore modify rumen fermentation dynamics by inhibiting growth of Gram-positive bacteria, including lactate-producing rumen bacteria such as *Streptococcus bovis* (10), reducing ruminal lactate production and the risk of acidosis. Moreover, studies reported that monensin reduces ruminal protein degradation and decreased microbial protein synthesis (11, 12), due to inhibitory effects on hyper-ammonia-producing bacteria (13). Despite the beneficial effects of monensin, the use of antibiotics and growth promoters has raised the concern about the risk of these products in increasing bacterial resistance to antibiotics and, consequently, possible risks to human health (14). However, these mechanisms of resistance are not fully understood, since the genes responsible for ionophore resistance in ruminal bacteria have not been identified (15).

As a result, there is a search for antibiotic replacements to modulate ruminal fermentation to control acidosis and increase animal performance (16). An alternative to antibiotics is passive immunization with polyclonal antibodies preparations (PAP) against specific groups of ruminal bacteria, such as lactate-producing ruminal bacteria (*Streptococcus bovis*) and bacteria related to liver abscesses (*Fusobacterium necrophorum*). In this context, the use of PAP against lactate-producing ruminal bacteria have led to a reduction in the concentration of rumen lactate (17–19), and also in the control of acidosis in animals during the rapid transition to a high-concentrate diet (16, 20).

Recent ruminal metabolism studies with PAP were conducted under the most extreme condition of abrupt transition to high-concentrate diets (18, 20). However, most experiments carried out with acidosis induction for the evaluation of additives were designed to monitor rumen fermentation in short periods (between 1 and 2 d; 3.5–7 g PAP/d (18, 20). In this context, there is a lack of information on the persistence of action of feed additives, as well as the administration time necessary to reduce the negative effects of this nutritional disorder on ruminal fermentation. Furthermore, this allows the comparison of PAP with other additives, such as MON, under these conditions of the longer evaluation period.

Therefore, it was hypothesized that, in a situation of induction of ruminal acidosis, MON reduces the flow of microbial protein to the intestine. Furthermore, PAP would be effective in preventing lactic acidosis after an abrupt change to high-concentrate diet. Thus, the present study aimed to evaluate the spray-dried multivalent polyclonal antibody preparation against lactate-producing bacteria (*Streptococcus bovis, Fusobacterium necrophorum*, and *Lactobacillus ssp*.) as an alternative to monensin to control ruminal acidification in cow fed high concentrate diets.

## 2. Materials and methods

All protocols and procedures followed in this study were approved by the São Paulo State University Ethical Committee for Animal Research (CONCEA, 2013), São Paulo State University, Botucatu campus, Brazil.

### 2.1. Polyclonal antibody preparation

Polyclonal antibodies were produced by CAMAS Inc. (Le Center, MN, USA). The commercial product contains 39.5% immunoglobulins against *Streptococcus bovis* (ATCC 9809), 17.6% against *Lactobacillus ssp*. (ATCC 4356; 14917; 9649 and 7469), 13.20% against *Fusobacterium necrophorum* (ATCC 27852), 17.6% against *E. coli* O157:H7, and endotoxins. For PAP preparation, the procedures were similar to those described by DiLorenzo et al. (17), with the exception that a multivalent PAP was tested, rather than antibodies to specific organisms. *Streptococcus bovis* and *Lactobacillus ssp*. are the main lactic acid-producing bacteria during the process of acidosis; *Fusobacterium necrophorum* is strongly associated with liver abscess in cattle; while *E. coli* is a commensal in the rumen and can be opportunistically pathogenic in humans. Endotoxins are the lipopolysaccharides that are present in the walls of gram-negative ruminal bacteria, being released in situations of bacterial death. The PAP in solid form was obtained by spray drying and was maintained in hermetically sealed packages Protected from heat light during the experimental period.

### 2.2. Animals and experimental facilities

The experiment was conducted at the School of Veterinary Medicine and Animal Science at the University of São Paulo (USP), Campus of Pirassununga, São Paulo, Brazil. Nine non-pregnant and non-lactating Holstein cows with an average live weight of 677 ± 98 kg previously fitted with ruminal cannulas were used. The animals were housed in individual stalls (Stall size: 1.5 m bunk space per 9 m. 13.5 m2 per animal) with sand bedding, feed bunk, and access to drinking water. The facility had fans suspended from the ceiling that was turned on automatically during the hottest hours of the day, to mitigate the effects of ambient temperature.

### 2.3. Experimental design and treatments

An incomplete Latin square design was used, divided into two experimental periods of 20 d each (d−5 to d 14). The choice for only two experimental periods is attributed to the attempt to avoid diluting the challenge effect of high-concentrate diets by potential metabolic memory in an attempt to avoid large intakes of concentrate. Cattle were submitted to the following treatments: (1) Control (CTL); (2) Monensin (MON); (3) Polyclonal Antibody Preparations (PAP) against *Streptococcus bovis, Fusobacterium necrophorum*, and *Lactobacillus ssp*. The PAP and Monensin were inserted through the ruminal cannula twice a day, before each meal and inside envelopes made of absorbent paper, between days 0 and 14 of each experimental period. The MON was administered at a dose of 300 mg/d, which corresponds to 3 g/d of the commercial product Rumensin (Elanco Animal Health, Indianapolis, IN). This commercial product contains 10% sodium monensin per kilogram of product. The PAP (CAMAS Inc, Le Centre, MN, USA) was administered at a dose of 3 g/d (corresponding to 10 mL of liquid product).

The experiment had a total duration of 55 d, divided into two periods of 20 d each (d−5 to d 14), with an interval of readaptation to the roughage diet of 15 d between periods. The washout period was used to reestablish normal ruminal pH conditions. During d−5 to−1, the cows received only a roughage diet, composed of sugarcane, urea (1.4% DM), and a mineral supplement to supply the following levels of crude protein (CP), NDF, and ADF (DM basis): 14.05, 43.98, and 22.18%, respectively. From d 0 to d 14, a 74% concentrate diet was offered (Table 1), composed of sugarcane, high-moisture corn silage, soybean meal, and vitamin and mineral premix.

**Table 1:** Feed ingredients and chemical composition of the experimental diet.

**Item**	**Experimental diet**
**Ingredients, % of DM**	
Sugarcane, fresh and chopped	26.5
High-moisture corn silage	53.6
Soybean meal	17.9
Vitamin and mineral premix^a^	1.0
Calcitic limestone	1.0
**Nutrient content**	
Dry matter (%)	55.0
Crude protein (%DM)	14.8
Rumen degradable protein (% CP)	72.0
Rumen undegradable protein (%CP)	28.0
Neutral detergent fiber (% DM)	23.3
Physically effective neutral detergent fiber (% DM)^b^	13.0
Non-fiber carbohydrates (% DM)	55.0
Starch (% DM)	32.6
TDN (% DM)	80.0
Ca (%DM)	0.6
P (%DM)	0.4

a Composition of vitamin and mineral premix per kilogram of product: 230 g of Ca, 90 g of P, 15 g of S, 20 g of Mg, 48 g of Na, 100 mg of Co, 700 mg of Cu, 2.000 mg of Fe, 80 mg of I, 1.250 mg of Mn, 20 mg of Se, 2.700 mg of Zn, 900 mg of F (maximum), 200.000 UI of vitamin A, 60.000 UI of vitamin D3, 60 UI of vitamin E.

b Estimated by equations according to CNCPS, Cornell version 5.0.40.

Between d−5 to d 14, the following variables were measured: individual dry matter intake; ruminal pH; ruminal concentration of total lactate and short-chain fatty acids (SCFA); NH_3_-N concentrations, and microbial protein synthesis.

Before the two experimental periods, ~20 kg of rumen content was extracted from each animal and these portions were mixed. After this procedure, the same amount of rumen content, removed and already mixed, was returned to each animal. This procedure aimed to homogenize the ruminal microbial population before the application of the experimental treatment.

### 2.4. Nutritional management

Diets were offered twice a day, at 800 h and 1,600 h. The experimental diet was administered as a total mixed ration (TMR), with a roughage: concentrate ratio of 26:74, in which the roughage source used was fresh sugarcane (2.9% CP, 47.48% NDF, and 25.68% ADF, DM basis) chopped with a theoretical mean particle size of 1.14 cm (21). The concentrate was composed of soybean meal (44.12% CP, 20.57% NDF, and 7.30% ADF, DM basis), and high-moisture corn silage (7.91% CP, 6.22% NDF, and 3.31% ADF, DM basis; Table 1). The DM, mineral matter (MM), CP, ether extract (EE), calcium, and phosphorus analyzes were performed according to AOAC (22), while the NDF corrected for ash and ADF were performed according to Van Soest et al. (23). For NDF analysis, α-amylase and urea were added. The starch concentration was carried out according to Pereira and Rossi Jr. (24), in which extraction of carbohydrates was performed according to Hendrix (25). The diet was formulated according to the NRC (26) and evaluated in the Cornell Net Carbohydrate and Protein System (CNCPS program, version 5.0.40) (27).

### 2.5. Dry matter intake

To evaluate dry matter intake in kg (DMI), DMI expressed as % of BW (DMI % BW), DMI expressed as g/kg of metabolic weight (DMI g/kg BW0,75), the amount of diet offered and refused were collected and weighed daily, from d−5 to d 15. All feed bunks were examined every morning. If there was no feed remaining, the amount offered was raised by 10%. If up to 10% remained, the amount of feed offered was not changed and if the surplus was >10%, the feed offered was reduced by 10%. Additionally, the fluctuation of dry matter intake (DMIF) was calculated for each animal, as the difference in dry matter intake between consecutive days, according to the methodology proposed by Bevans et al. (28), as follows:


$$\text{DMIF = }[\frac{\left(\text{DMI current day }-\text{ DMI previous day}\right)}{\text{DMI previous day}}]{\;}^{*}\text{100}$$

The DMIF was performed between days−1 and 3 of each experimental period.

### 2.6. Ruminal fermentation parameters

At each daily collection, at least 500 mL of rumen content was removed at three different points of the rumen (through an electric vacuum pump), which were returned to the rumen-reticulum after collecting the appropriate aliquots for determination of lactate, NH_3_-N, and SCFA molar concentrations. The collections were carried out daily, from d−5 to d 14 at 1,100 h (3 h after the morning feeding, carried out at 800 h) (29). Immediately after collection, 100 mL of rumen fluid was used for pH determination in a portable digital potentiometer (HANNA instruments HI8424), calibrated with pH 4.0 and pH 7.0 buffer solutions. Regarding the determination of the days on which the animals presented acidosis (DEA), according to definitions created by several authors (6, 9, 30), sub-acute acidosis was considered to occur when the pH was ≤ 5.6. So, the number of days in which the ruminal pH of each animal was pH < 5.66 was counted. The adoption of the second decimal place is due to the sensitivity of the pH measuring device. Thus, pH values starting at 5.66 were considered pH 5.7 and discarded as acidotic pH. Only the post-challenge experimental phase was accounted for (d 0 to d 14), and the results were expressed as a percentage of this phase.

To determine the ruminal total lactate, 2 mL of rumen fluid was placed in test tubes and subsequently measured by the colorimetric technique according to Pryce (31). For short-chain fatty acid (SCFA) analyses that included acetate, propionate, and butyrate, a fraction of ~100 mL of rumen content was centrifuged at 2000 x g for 20 min; 2 mL of the supernatant was added to 0.4 mL of formic acid and frozen at−20 °C for further analyses, according to Erwin et al. (32). The SCFA were measured by gas chromatography (Thermo Scientific^®^, model Focus GC) with an automatic sample injector (Thermo Electron Corporation^®^, model AS-3000) equipped with a 2 m long, and 1/5” diameter glass column was used, packed with Carbopack B-DA/4% Carbowax^®^ 20M 80-120 (Supelco^®^) and flame ionization detector (FID) maintained at 270°C. The gas chromatograph oven was maintained at 190°C during the analysis and the injector temperature was 220°C. The carrier gas was high-purity H2, maintained in a flow of 30 mL/min. The number of repetitions per sample was the one necessary for the difference between readings to be <5%.

To determine the concentration of NH_3_-N, fractions of 2 mL of rumen fluid were placed in test tubes containing 1 mL of 1N sulfuric acid solution and stored under refrigeration until the analysis by colorimetry (Kjeltec 2300 Analyzer Unit, Tecator, Hoganas, Sweden), according to the method described by Kulasek (33) and adapted by Foldager (34).

### 2.7. Estimation of microbial protein synthesis in the rumen

Analyses to determine microbial protein synthesis were performed at the Laboratory of Animal Biochemistry and Physiology of the VNP-FMVZ/USP, based on the quantification of urinary purine derivatives (PD), according to the methodology described by Valadares et al. (35) and Rennó (36), considering the absorption of purines from the formula suggested by Verbic et al. (37).

Urine samples (50 mL, spot sample) were collected from all animals on d−3; 3, and 14 of each experimental period, ~3 h after feeding. The urine was filtered and 10 ml aliquots were immediately diluted in 40 mL of 0.018M sulfuric acid to avoid bacterial destruction of purine derivatives and uric acid precipitation, then stored at −15 °C for further analysis of allantoin and acid. uric. A pure urine sample was stored for the determination of total nitrogen compounds, urea and creatinine.

Creatinine concentrations were determined by commercial kits (Laborlab^®^), using an enzymatic reaction in a spectrophotometer (SBA-200 Celm^®^). The total daily urinary volume was estimated by dividing the daily urinary excretions of creatinine by the observed values of creatinine concentration in the urine of the spot samples according to Oliveira et al. (38).

The daily urinary excretion of creatinine was estimated from the established mean daily excretion of 24.05 mg/kg body weight for dairy cows (39). Thus, with the average daily excretion of creatinine and the concentration of creatinine (mg/dL) in the spot urine sample, the total daily volume of urine, in liters per cow, was estimated. The levels of allantoin and uric acid in the urine were determined by the colorimetric method, according to the methodology of Fujihara et al. (40), described by Chen and Gomes (41).

The total excretion of PD was calculated as the sum of allantoin and uric acid excreted in the urine, expressed in mmol/day. Absorbed microbial purines (AP, mM/day) were calculated from the urinary excretion of purine derivatives (PD, mM/day), using the equation:


$$\text{AP = }(\text{PD 0}.\text{236}{\;}^{*}{\text{BW}}^{\text{0}.\text{75}})\text{/0}.\text{84}$$

where 0.84 is the recovery of purines absorbed as purine derivatives and 0.236 is the endogenous excretion of PD (42).

Absorbed microbial purines were also assessed, considering the endogenous excretion of 0.512^*^BW^0.75^ and the recovery of 0.70 found by Gonzalez-Ronquillo et al. (43). Microbial protein synthesis (Pmic, g of N/day) was calculated based on the AP (absorbed microbial purines, mM/day), using the Equation (41):


$$\text{Pmic = }({\text{70}}^{*}\text{AP})\text{/}(\text{ 0}.{\text{83}}^{*}\text{0}.{\text{134}}^{*}\text{1000})$$

where 70 is the N content in the purines (mgN/mol); 0.134, the purine N: total N ratio in bacteria (35); and 0.83, the intestinal digestibility of microbial purines. To obtain the microbial crude protein synthesis, the Pmic data were multiplied by the Kjeldahl factor of 6.25.

### 2.8. Statistical analysis

The experimental design was an incomplete Latin square. Data were analyzed by Statistical Analysis System software (SAS version 9.2; SAS Inst., Inc., Cary, NC, USA), in which the model included the effects of treatments as fixed, and period and animal as random. Before the analysis of variance, the normality of the residuals was verified by the SHAPIRO-WILK Test (PROC UNIVARIATE), and the variances were compared by the “F” Test. Data (dependent variable) that did not meet these premises were submitted to logarithmic [Log (X+1)] or square root [SR (X+1/2)] transformation. The original or transformed data, when the latter procedure was necessary, were subjected to analysis of variance that separated the effects of treatments and period as sources of variation, plus the factor repeated measures over time, referring to the different sampling days. Such analysis was performed using the MIXED procedure of SAS. The effect of time analysis was only reported when the interaction between time and treatment effects was significant. The differences between means were performed using the Tukey test. Effects were considered significant at *P* < 0.05.

## 3. Results

### 3.1. Dry matter intake

**Figure 1:**
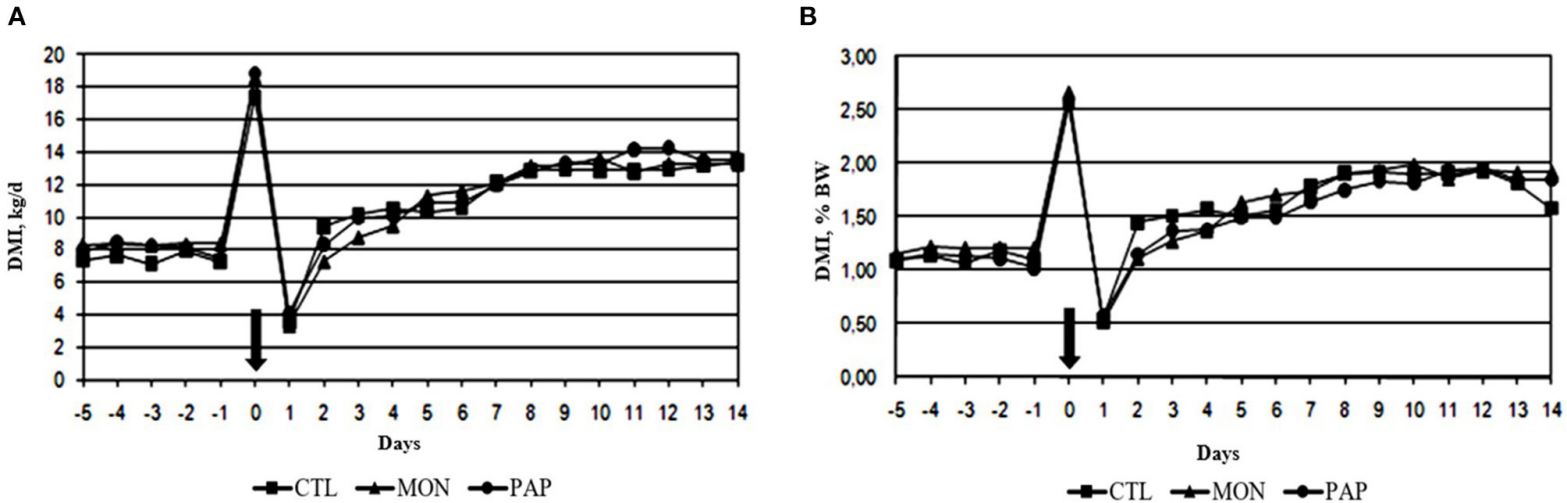
Dry matter intake expressed in kg [DMI, **(A)**] and as a percentage of body weight [DMI %BW, **(B)**] of cows induced to ruminal acidosis. The bold arrow represents the change from the 100% forage diet to the 74% concentrate diet containing the treatments.

No interactions were found between day and treatment for DMI (*P* = 1.00), DMI % BW (*P* = 0.99), and DMI g/kg BW0,75 (*P* = 0.99). The administration of feed additives *via* ruminal cannula did not result in changes (*P* > 0.11) in DMI when compared to the control (Tables 2, 3). However, a day effect (*P* < 0.01) was found for all dry matter intake variables mentioned above. There was a large peak of intake on d 0 (18.29 kg or 2.60% of BW), the day when the experimental diet was started, followed by a large decline on d 1 (3.67 kg or 0.54 % of BW; Figure 1). From d 2, DMI showed an increasing pattern (8.34 kg or 1.23 % of BW) and stabilized around d 8 (12.96 kg or 1.86 % of BW) for all treatments. The mean difference in intake between the forage diet on d−1 and the experimental diet on d 0 was 10.61 kg. On the other hand, cows ingested 14.63 kg less feed on d 1, compared to d 0. Additionally, a decrease in the variation of DMI was observed from d 2 (4.67 kg) and d 3 (1.29 kg), which corroborates the increasing pattern found in DMI and DMI % BW.

**Table 2:** Dry matter intake in kg (DMI, kg/d), DMI as a percentage of body weight (DMI, %BW), and DMI based on metabolic BW (DMI, g/kg BW0.75) of cows induced to ruminal acidosis receiving polyclonal antibody preparations (PAP) or monensin (MON).

	**Treatments**					
**Item**	**CTL**	**MON**	**PAP**	**Mean** ^a^	**SEM** ^b^	***P*** **-value**
DMI, kg	10.71	10.87	11.04	10.88	0.20	0.50
DMI, % BW^c^	1.59	1.58	1.51	1.56	0.03	0.11
DMI, g/kg BW^0, 75^	80.90	80.67	78.48	80.02	1.43	0.44

a Mean across treatments: CTL, Control; MON, Monensin; PAP, Polyclonal Antibody Preparation.

b SEM, standard error of mean.

c BW, body weight = 677 ± 98 kg.

**Table 3:** Fluctuation in dry matter intake (DMIF, kg) of cows induced to ruminal acidosis receiving polyclonal antibody preparations (PAP) or monensin (MON).

	**Treatments**					
**Day** ^a^	**CTL**	**MON**	**PAP**	**Mean** ^b^	**SEM** ^c^	***P*** **-value**
0	10.16	10.22	11.45	10.61	0.81	0.79
1	−13.88	−15.25	−14.76	−14.63	1.16	0.90
2	5.88	3.95	4.19	4.67	0.56	0.33
3	0.72	1.47	1.68	1.29	0.62	0.81
Overall	0.72	0.10	0.64	0.49	1.18	0.82

a Represents the difference in DMI (kg) between consecutive days.

b Mean across treatments: CTL, Control; MON, Monensin; PAP, Polyclonal Antibody Preparation.

c SEM, standard error of mean.

### 3.2. Rumen fermentation

**Figure 2:**
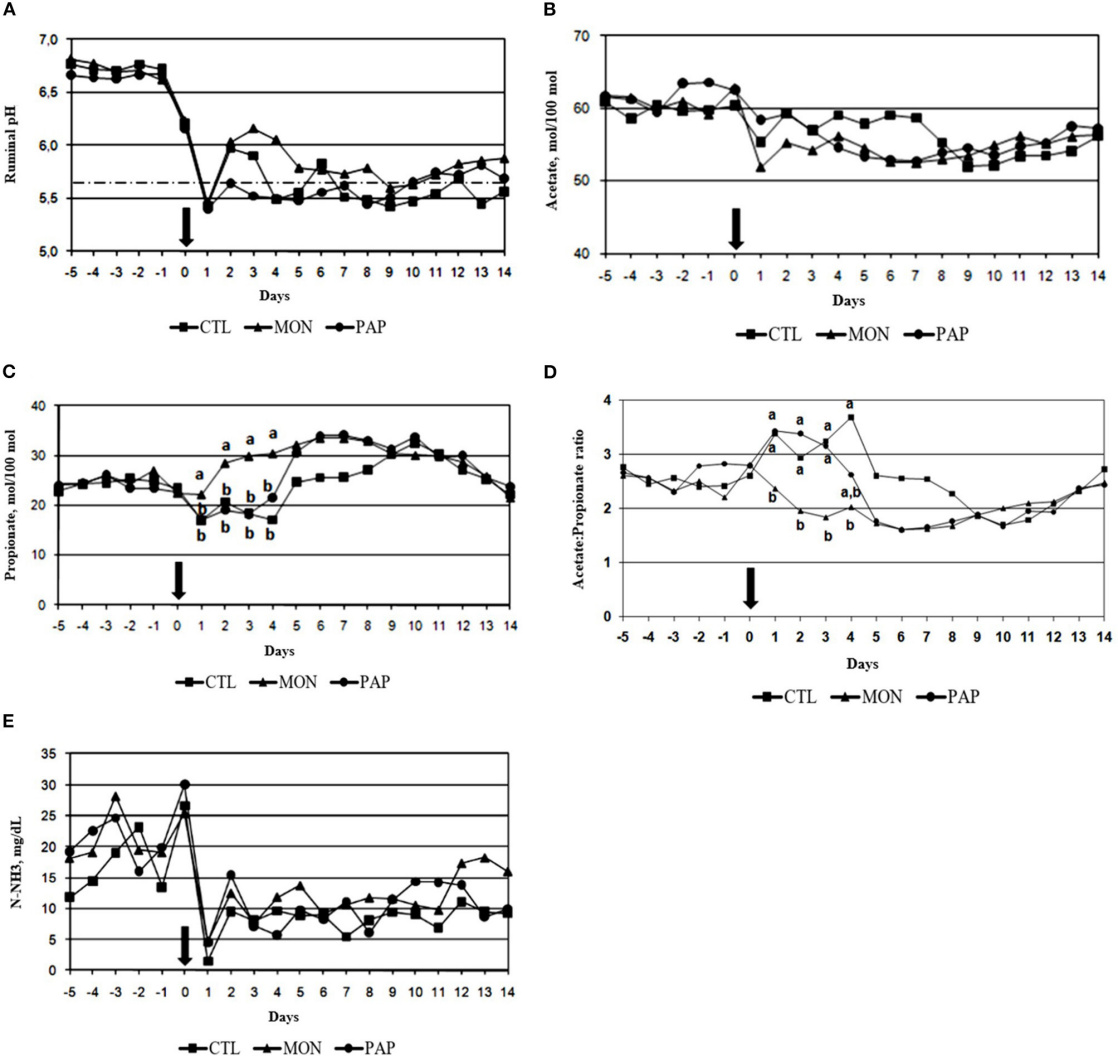
Ruminal pH **(A)**, acetate [% molar proportion, **(B)**], propionate [% molar proportion, **(C)**], acetate: propionate ratio **(D)**, and N-NH3 concentration **(E)** of cows induced to ruminal acidosis receiving polyclonal antibodies (PAP) or monensin (MON). The bold arrow represents the change from the 100% forage diet to the 74% concentrate diet containing the treatments.

No interaction was observed between day and treatment (*P* = 0.19) for rumen pH 3 h after feeding. Intraruminal addition of MON resulted in higher pH (*P* < 0.01) at the third postprandial hour compared to the other treatments (MON = 6.06 vs. CTL = 5.91 and PA*P* = 5.89; Table 4). Additionally, a day effect was found (*P* < 0.01) for rumen pH, where the decline in pH commenced on day 0 and the maximum decline was on day 1 (Figure 2A) regardless of treatments.

**Table 4:** Ruminal fermentation variables of cows induced to acidosis and receiving polyclonal antibodies preparations (PAP) or monensin (MON).

	**Treatments**					
**Item**	**CTL**	**MON**	**PAP**	**Mean** ^1^	**SEM** ^2^	***P*** **-value**
pH	5.91^b^	6.06^a^	5.89^b^	5.95	0.03	<0.01
DEA^3^, %	60.00	32.22	58.89	50.37	6.93	0.14
Lactate, m*M*	0.23	0.21	0.25	0.23	0.009	0.23
Acetate, mol/100 mol	57.08	56.44	57.37	56.97	0.29	0.33
Propionate, mol/100 mol^*^	24.48^b^	28.08^a^	26.28^b^	26.28	0.45	0.04
Butyrate, mol/100 mol	18.43^a^	15.42^b^	16.35^b^	16.74	0.29	<0.01
Total SCFA, m*M*	115.81^b^	115.04^b^	120.17^a^	117.00	1.23	0.02
NH_3_-N, mg/dL	11.20^b^	14.74^a^	13.64^a^	13.19	0.53	<0.01

1 Mean across treatments: CTL, Control; MON, Monensin; PAP, Polyclonal Antibody Preparation.

2 SEM, standard error of mean.

3 DEA = percentage of days from the challenge with the high-concentrate diet whose pH measured at the third postprandial hour was <5.66.

* Significant interaction between day and treatment (P < 0.01).

a, b Values within a row with different superscripts differ (*P* < 0.05).

There was no interaction between day and treatment (*P* = 0.78) for rumen lactate. The molar concentration of lactate remained low throughout the experimental period (0.23 mM). The molar concentration of SCFA was higher in cows treated with PAP (*P* = 0.02) but there was no interaction between day and treatment (*P* = 0.20). Furthermore, a treatment effect was observed (*P* = 0.02), in which cows receiving PAP presented the greatest concentration. Similarly, a day effect was detected (*P* < 0.01), in which the total SCFA increased from ~90 mM in the pre-challenge period to a maximum value of 135 mM on d 1, stabilizing around 129 mM from d 8 (data not shown).

The interaction between day and treatment was not significant (*P* = 0.38) for the molar concentration of acetate. Additionally, neither feed additive influenced acetate concentrations (*P* = 0.33; Table 4). There was a time effect (*P* < 0.01) for the molar proportion of this SCFA (Figure 2B), where the molar proportions of acetate went from ~60% to around 55% on d 1, after the abrupt change to the experimental diet.

There was an interaction between day and treatment (*P* < 0.01; Figure 2C), in which an increase in the molar proportions of propionate was observed in cows receiving MON during the first 4 days following the challenge, an increase of 78% on d 4 when compared to control (MON = 30.42 vs. PA*P* = 21.63 and CTL = 17.07 mM on d 4). An interaction between day and treatment (*P* = 0.01) was also observed for the acetate/propionate ratio, in which MON reduced this proportion in the three first days following the challenge (Figure 2D).

There was no interaction between day and treatment (*P* = 0.25) for the molar concentration of butyrate, but both feed additives were effective (*P* < 0.01; Table 4) in decreasing ruminal butyrate (MON = 15.42 and PA*P* = 16.35 vs. CTL = 18.43).

The interaction between day and treatment was not significant (*P* = 0.90) for NH_3_-N concentration. However, a treatment effect was found (<0.01), in which animals treated with additives had higher concentrations than CTL animals (MON = 14.74 and PA*P* = 13.64 vs. CTL = 11.20). Moreover, the day effect was significant (*P* < 0.0001), where the animals went from a concentration of around 19 mg/dL when fed a forage diet, to 27 mg/dL on d 0, 3.56 on d 1, and remaining around 12 mg/dL (with large variations) until the end of the experiment (Figure 2E).

### 3.3. Microbial protein synthesis

**Figure 3:**
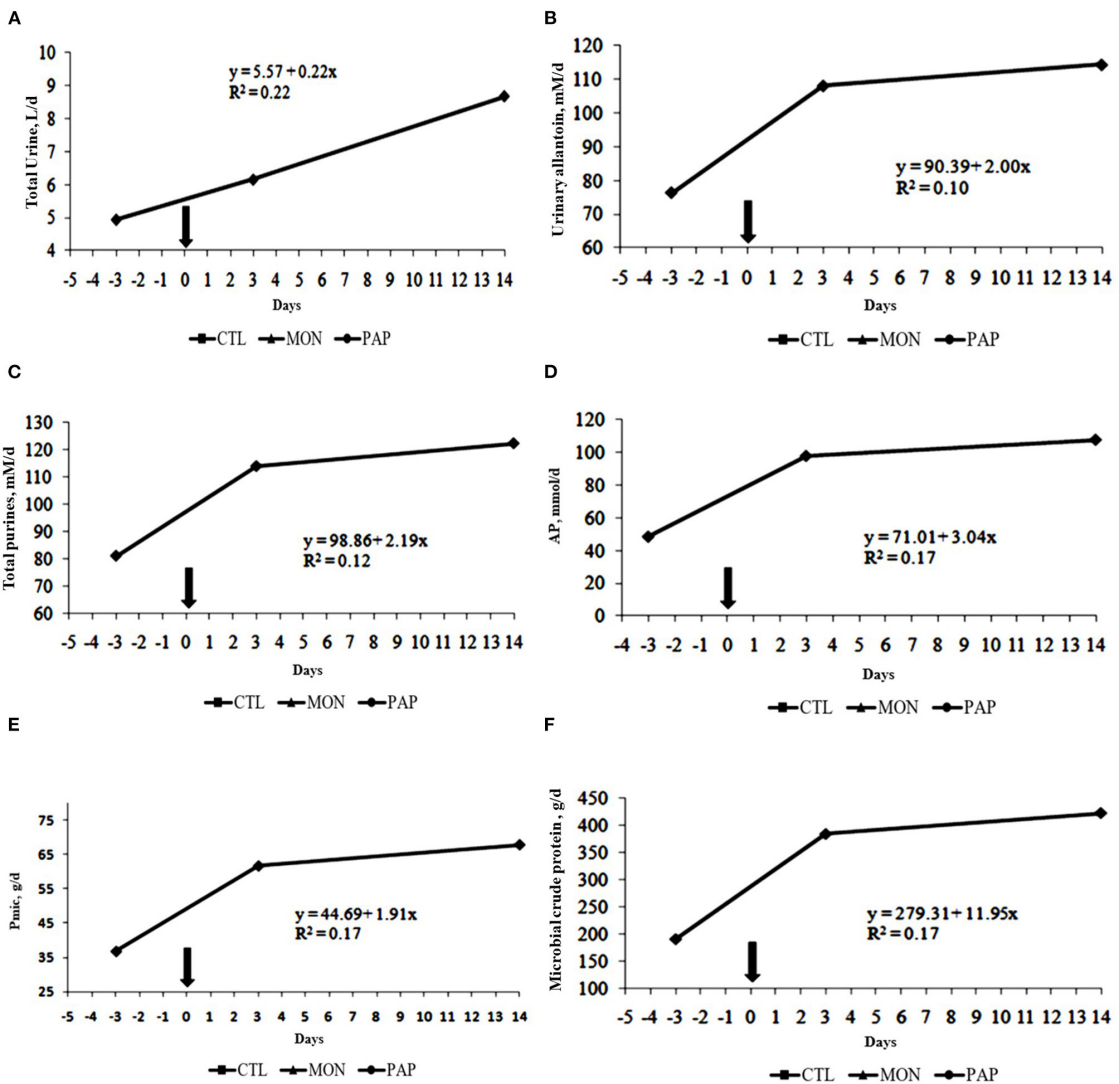
Total urine excreted **(A)**, urinary allantoin **(B)**, total purines **(C)**, and absorbed microbial purines **(D)**, microbial-N **(E)** and microbial crude protein **(F)** of cows induced to ruminal acidosis. The bold arrow represents the change from the 100% forage diet to the 74% concentrate diet containing the treatments.

No interactions were observed between day and treatment for any of the evaluated experimental variables (*P* > 0.05). Moreover, the treatments with feed additives did not result in significant differences (*P* > 0.05, Table 5) for any experimental variable related to microbial protein synthesis (Figure 3).

**Table 5:** Microbial protein synthesis in cattle induced to ruminal acidosis receiving polyclonal antibodies (PAP) or monensin (MON).

	**Treatments**					
**Item**	**CTL**	**MON**	**PAP**	**Mean** ^a^	**SEM** ^b^	***P*** **-value**
Total urine excreted, L/day	6.57	7.76	5.72	6.70	0.47	0.12
Urinary allantoin, mM/day	104.72	106.51	89.92	100.38	6.20	0.41
Urinary uric acid, mM/day	7.04	6.40	5.81	6.42	0.59	0.61
Total purines, mM/day	111.74	112.90	95.75	106.80	6.41	0.38
Allantoin in relation to total purine, %	92.80	94.74	93.58	93.58	0.54	0.31
Absorbed purines, mM/day	95.58	90.0	74.66	86.68	7.35	0.42
Microbial protein synthesis (Pmic), g/day	60.15	60.90	46.99	56.01	4.76	0.43
Microbial crude protein, g/day	375.93	354.03	293.66	340.95	28.92	0.43

a Mean across treatments: CTL, Control; MON, Monensin; PAP, Polyclonal Antibody Preparation.

b SEM, standard error of mean.

## 4. Discussion

The results of the current study indicate that monensin was effective in controlling subacute ruminal acidosis in cattle abruptly shifted to a high-concentrate diet, and also in improving rumen fermentation by altering SCFA molar proportions. In addition, the acidosis challenge induced by the abrupt increase in diet energy levels was caused by the accumulation of SCFAs. As a result, PAP was not effective in controlling acidosis, which can be explained by the fact that this product targets the control of lactate-producing bacteria. However, it is worth mentioning that the experimental design, as well as the protocol used in the present study, which included a simultaneous increase in diet density and different treatments inserted through the rumen cannulae, may have influenced the results. Since cows get used to consecutive acidosis challenges, an incomplete Latin square was adopted. Despite of these limitations, and considering the complexity of acidosis induction studies, the experimental protocol used in the study was efficient for inducing acidosis.

The reduction in DMI and increases in feed efficiency in animals fed high-concentrate diets in response to MON supplementation are well documented in the literature (8, 9). Similarly, reductions in intake in animals receiving MON have been found in comparative performance studies with PAP (44, 45). However, in the present study, MON did not reduce the DMI after an abrupt transition to high-concentrate diets, and this corroborates the absence of difference in DMI reported by Rodrigues et al. (46) where they found no reduction in dry matter intake in animals that received MON, also compared to PAP. From a metabolic point of view, the evaluation of the intake behavior of animals fed high-concentrate diets with MON may provide better information than the simple quantification of the total daily intake, because this negative effect of MON on DMI is related to a lower DMI per meal (47, 48), which consequently leads to an increase in the number of meals without changing the total amount of dry matter ingested daily (49, 50). In this context, this is the best mode of action for a feed additive to prevent subacute acidosis, associated with increased concentrations of SCFA (9), and, consequently, the control of dry matter intake prevents excessive fermentation of high starch content. These facts may explain the faster recovery of pH to normal rumen conditions in animals treated with MON in the present study.

However, the low DMI observed on day 1 may be explained by the increase in rumen osmolarity, as a result of reduced absorption and an increase in substances that contribute to an increase in osmolarity, such as glucose, SCFAs, and lactate, may lead to an influx of fluid from the blood into the rumen and, consequently, decrease DMI (51). In the present study, the greatest fluctuations in DMI occurred between day−1 and day 1, where the lowest average daily pH (5.43) was observed on day 1. Increases in DMI variation have been identified as an indicator of subacute acidosis (52, 53). However, the fluctuations decreased over the days, and instead of a cyclic pattern, as would be expected in animals that experienced ruminal acidosis, an increase in DMI was observed from day 2 onwards. The large amount of feed consumed by cows on the day of the challenge (d 0) can be explained by the fact that ruminants show a preference for feeds or diets that compensate for nutrient deficiencies (54). During the pre-challenge phase, cows were fed a high-forage diet. This may have increased the avidity for the concentrate after the challenge, increasing the DMI from day 2.

Furthermore, by evaluating pH and lactate concentration, it is possible to classify the acidosis caused by the challenge in this study as subacute (9). However, it is worth noting that in the present study, a single sample was collected 3 h after feeding on each day of the experimental period (29), but a recent study reported that rumenocentesis should be performed in the late afternoon or evening to maximize the probability of detecting animals with pH values below the threshold level (55, 56). Subacute ruminal acidosis (SARA) is characterized by a decline in ruminal pH below 5.8 or 5.6 (57). However, the diagnosis of SARA should not be made based on rumen pH alone, but in combination with symptoms to make SARA identification more accurate and feasible, including fecal consistency, rumen motility, and inflammatory markers (57). Nevertheless, there is not complete agreement on the etiology and symptoms of SARA (57, 58). In recent years, the development of sequencing technologies has enriched the study of SARA by expanding the understanding of the rumen microbiota (59). Changes in the structure and function of the rumen microbiota have been reported during SARA, including decreased bacterial richness and diversity, decreased relative abundance of fibrolytic bacteria and increased levels of amylolytic bacteria, and increased levels of propionate and total SCFA (59, 60). In the present study, feeding MON was more effective in minimizing reductions in rumen pH when compared to PAP and CTL. It is well documented in the literature that MON improve feed efficiency by reducing dry matter intake (47), reducing ruminal lactate production because this ionophore shifts the rumen microbial population by inhibiting growth of Gram-positive bacteria, including lactate-producing rumen bacteria such as *Streptococcus bovis* (10). However, considering only the average pH from day 0 to day 14, it was observed that cattle fed MON had a minimized risk of ruminal acidosis (pH 5.84), whereas cows from the other two treatments presented, on average, acidotic pH (pH 5.60).

In addition, the high-grain diet challenge resulted in some clinical manifestations, which are indicative of SARA, such as diarrhea in all animals on day 1. The change in feces could be due to the large flow of readily fermentable carbohydrates from the rumen to the intestine, causing excessive fermentation in these organs (61). Also, the high osmolarity promoted by the experimental diet described in animals with subacute ruminal acidosis, may retain fluid in the lumen and alter fecal consistency (62). The increase in total SCFA concentration in the rumen of cows fed PAP was not sufficient to reduce ruminal pH to a level below that of cows fed no feed additive. Therefore, feeding PAP may play a role in controlling rumen acidification during an abrupt change from a high-forage to a high-concentrate diets.

In this context, the effect of MON in modulating the lactate-producing bacteria population *in vivo* and *in vitro* in acute acidosis situations is well described in the literature (51). The microbiological changes in acute and lactic acidosis are also well documented in the literature (9), but very little is known about the changes that occur in ruminants with subacute acidosis (63). This may be because MON is a feed additive with a broad-spectrum of activity as it reduces the rumen fermentation rate, which may be explained by the effect of treatment on the total SCFA concentration. It is worth mentioning that the lactate concentration was low in the present study. Based on this fact, the lactate levels in the ruminal fluid of cattle with subacute ruminal acidosis are usually not increased, which shows that the total concentration of SCFA is more important in subacute acidosis and the lactate concentration is more important in acute acidosis (6, 57, 64). Also, SARA may promote a gene expression change on rumen epithelium, due to an accumulation of intracellular cholesterol and its metabolites in response to a higher substrate supply (total SCFA). This could stimulate cell proliferation, increase membrane permeability, and induce epithelial inflammation, that eventually disrupts rumen homeostasis and negatively affects cow health (59).

In contrast, the lack of effect of PAP in controlling rumen pH may have been due to the high specificity of these antibodies. Since SARA was induced by high concentrations of SCFA, the favorable conditions for a true evaluation of this additive were not presented because the target microorganisms were mostly acid-tolerant bacteria. In clinical and sub-clinical acidosis, the rumen pH decreases to a point where cellulolytic bacteria are inhibited and lactate-producing bacteria predominate, particularly *Streptococcus bovis* and *Lactobacillus* sp. (65). However, the observation of the lack of results in studies performed with PAP in solid form (66) raises doubts about whether the loss of antibody activity occurs during the conversion of the liquid to solid phase. Cassiano et al. (18) reported that neither liquid nor powdered forms of PAP altered rumen acidosis variables in adapted or unadapted animals. In this context, further comparative studies between the two forms of product presentation and new drying techniques are recommended.

The effects of MON in manipulating rumen fermentation are due to changes in rumen microbial ecology (67, 68). The increase in the molar proportion of propionate, the decrease in the acetate/propionate ratio, and the decrease in the molar proportions of butyrate in animals treated with MON can be explained by the inhibition of the growth of the population of Gram-positive bacteria, which are sensitive to ionophores and produce mainly acetate, butyrate, H2, and formate (67). However, when the above changes are verified, a decrease in the molar proportion of acetate would be expected, an effect confirmed by others studies (67–69). Probably, the decrease in the molar proportions of acetate occurs in experimental situations where rumen fermentation is already stabilized, which was not the case in this study. Thus, given the abrupt change in diet and the start of administration of the treatments on the day of challenge, it may have taken some time for rumen bacterial community to change. In addition, low acetate/propionate ratios are desirable to some extent in cattle, to maintain the necessary daily weight gain (48, 70). However, low acetate/propionate ratios can lead to a decrease in DMI and weight gain in unstable situations (53). In dairy cattle, low acetate/propionate ratios may decrease milk fat and an increase in body condition scores (71).

Furthermore, SARA has been characterized as a condition of elevated SCFA concentration that can lead to a critical rumen pH because of the imbalance between the production and absorption of these acids (57, 72), causing reduced microbial protein synthesis (73). In the present study, no changes in microbial protein synthesis were observed in response to the treatments. Studies have reported a reduction in microbial protein flux into the gut and decreased efficiency of microbial protein synthesis in response to MON (74). However, these studies show results in situations in which fermentation would theoretically be stabilized, in contrast to the present study in which animals were induced to acidosis. A possible explanation for the lack of effect of the treatments would be that abrupt changes, promoted by both diet and additives, contributed to a greater growth of the ruminal microbial population, and shortened the time for MON to contain bacterial growth. As a result, a linear increase in purine derivatives excretion and an increase in microbial protein were observed during the experimental period, explained by the increase in energy density of the diet and the greater supply of rapidly fermentable carbohydrates (75).

Contrary to expectations, animals treated with both MON and PAP had higher concentrations of ruminal NH_3_-N. Both additives affect the population of *Streptococcus bovis*, a highly proteolytic gram-positive rumen bacterium (76) and therefore may indirectly affect rumen ammonia concentrations. In addition, MON may act on the partitioning of protein metabolism by decreasing the production of NH_3_-N in the rumen and increasing escape of dietary protein from ruminal degradation [increasing the passage rate of protein from 22 to 55%; (77) and (78), respectively]. However, despite decades of widespread use of this ionophore, the protein partitioning effect has never been fully explained (79), largely because of the observation that most isolated ammonia-producing rumen bacteria are Gram-negative (80). Chen and Russell (79, 81) were able to obtain three isolates of gram-positive bacteria (*Peptostreptococcus anaerobius, Clostridium sticklandii* and *Clostridium aminophilum*) with highly specific activities for the production of ammonia, which are also sensitive to MON in *in vitro* studies. However, further studies are needed to explain the increase in rumen NH_3_-N concentration in animals. However, it is noteworthy to mention that the protocol used in the present study, which involved a simultaneous increase in diet density and different treatments, may have influenced the results. Ammonia is the predominant base in the rumen (51); therefore the sharp decrease in the concentration of NH_3_-N observed on day 1 could be due to the low rumen pH, along with the decrease in DMI on that day. In addition, much of the NH_3_-N may have been incorporated by the bacteria, which may have had a high growth rate during this experimental period.

## 5. Conclusion

Monensin was effective in controlling subacute acidosis in cattle challenged with the abrupt transition to a high-concentrate diet and improved rumen fermentation by altering SCFA molar proportions. The PAP was not effective in controlling acidosis, which can be explained by the fact that this product targets the control of lactate-producing bacteria. However, the acidosis induced by the challenge was caused by the accumulation of SCFAs. Therefore, the real conditions for evaluation of this feed additive were not reached in this experiment, which opens the possibility of new studies under the conditions of lactic acidosis. It is worth mentioning that the protocol used in the present study, which included a simultaneous increase in diet density and different treatments, may have influenced the results.

## Data availability statement

The raw data supporting the conclusions of this article will be made available by the authors, without undue reservation.

## Ethics statement

The animal study was reviewed and approved by São Paulo State University Ethical Committee for Animal Research (CONCEA, 2013), São Paulo State University, Botucatu campus, Brazil.

## Author contributions

RP and DM: conceived and designed study, collected and complied, and analyzed data. CTM, CLM, JB, MA, and PR: provided intellectual input. RP, JS, and DM: provided intellectual input and drafted and edited manuscript. All authors contributed to the article and approved the submitted version.
